# Supplementary material for: The origin of suspended particulate matter in the Great Barrier Reef
Source: Nat Commun. 2023 Sep 12;14:5629. doi: 10.1038/s41467-023-41183-z (PMC10497579; doi:10.1038/s41467-023-41183-z)
Supplement: Supplementary file 1 — Supplementary Information [file 41467_2023_41183_MOESM1_ESM.pdf]

Supplementary Information for

The origin of suspended particulate matter in the Great Barrier Reef

Mohammad Bahadori[1, 2], Chengrong Chen[1, 2*], Stephen Lewis[3], Juntao Wang[4, 5], Jupei Shen[6], Enqing Hou[7], Mehran Rezaei Rashti[1, 2], Qiaoyun Huang[8], Zoe Bainbridge[3], Tom Stevens[3].

[1]Australian Rivers Institute, Griffith University, Nathan, Queensland 4111, Australia

[2]School of Environment and Science, Griffith University, Nathan, Queensland 4111, Australia

[3]Catchment to Reef Research Group, Centre for Tropical Water and Aquatic Ecosystem Research, James Cook University, Townsville, Queensland, Australia

[4]Hawkesbury Institute for the Environment, Western Sydney University, Penrith, New South Wales, Australia

[5]Global Centre for Land-Based Innovation, Western Sydney University, Penrith, New South Wales, Australia

[6]School of Geographical Sciences, Fujian Normal University, Fuzhou, PR China

[7]Key Laboratory of Vegetation Restoration and Management of Degraded Ecosystems, South China Botanical Garden, Chinese Academy of Sciences, Guangzhou 510650, China

[8]State Key Laboratory of Agricultural Microbiology, Huazhong Agricultural University, Wuhan, China

*Correspondence to: c.chen@griffith.edu.au

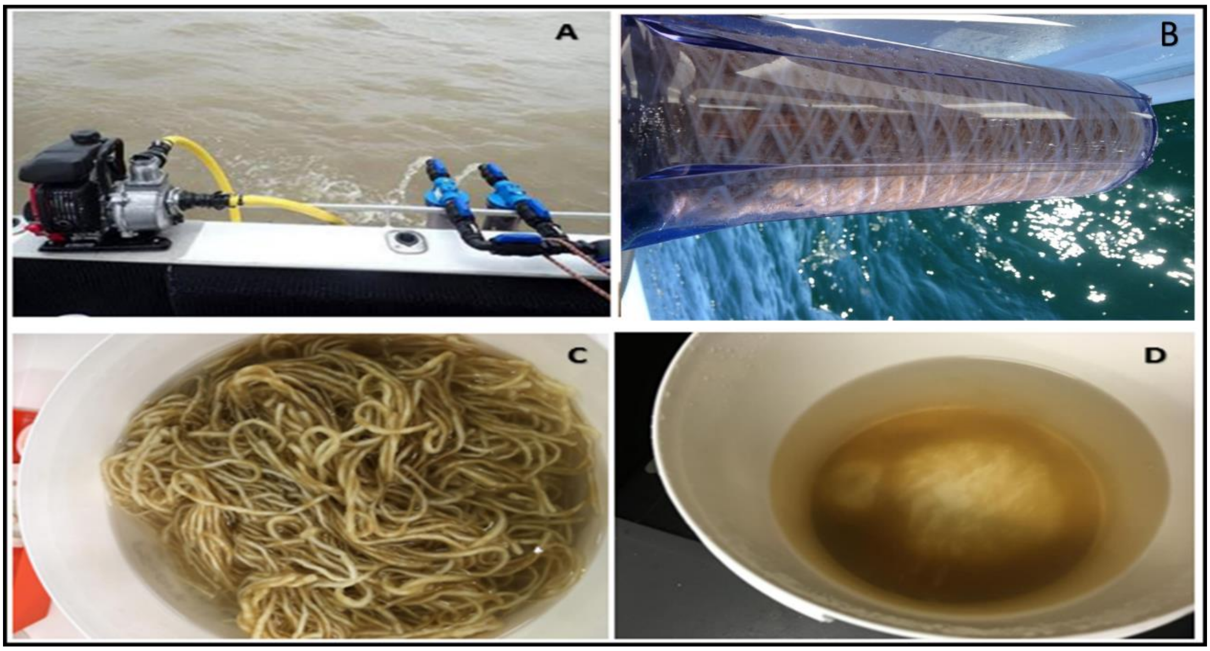

Fig. S1. The SediPump® system for collecting suspended particulate matter (SPM) from riverine, inner estuarine mixing zone (IEMZ) and outer estuarine mixing zone (OEMZ) of rivers. A and B: this sampling system allows large volumes of water (9,000 to 12,000 L) to be pumped through a 1 μm filter cartridge (Puretec® sediment filter cartridge-wound GW011) over a relatively short period (2 to 3 hours); C and D: The SPM retained in the filter carousel was recovered by cutting the string filter cartridge to release the SPM.

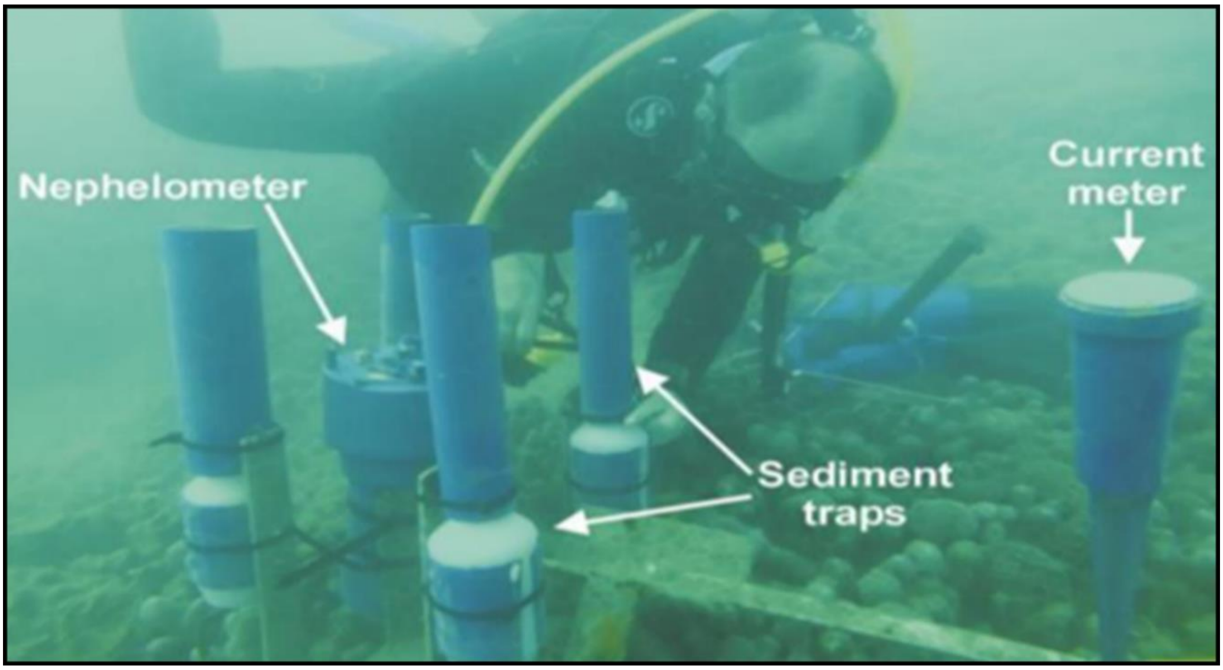

Fig. S2. Sediment traps (Sedisampler®) for collecting marine trap sediments (TS) surrounded by the nephelometer and current meter. Sediment traps were installed 50 cm off the seafloor along various sites on the inshore GBR shelf adjacent to the river sites to capture resuspension events and, during the wet season deployments, the influence of SPM delivered in riverine flood plumes.

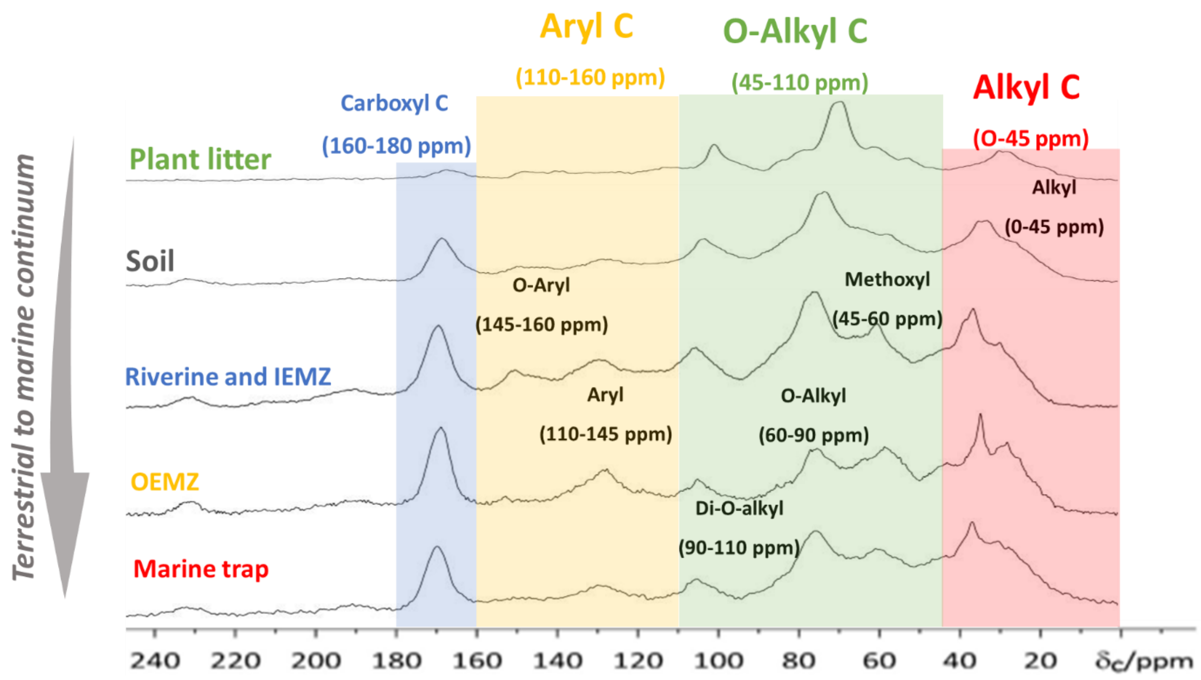

Fig. S3. Solid-state ^{13}C nuclear magnetic resonance (^{13}C NMR) spectra. This figure represents the spectra acquired for plant litter (75 MHz, 2,000 scans, 5 kHz), soil and suspended particulate matter (SPM) (75 MHz, 20,000 scans, 5 kHz). Assignments of the ^{13}C chemical shift regions are illustrated. Riverine and IEMZ: the SPM collected from riverine and inner estuarine mixing zone; OEMZ: the SPM collected from outer estuarine mixing zone.

Table S1. Changes in proportion of organic carbon functional groups during transportation of organic matter along the plant-soil-riverine-estuarine-marine continuum as identified by solid-state ^{13}C nuclear magnetic resonance (^{13}C NMR) spectroscopy.

Samples	n	O-Alkyl (%)	Alkyl (%)	Aryl (%)	Carboxyl (%)	A/O-A ratio
Plant litter	21	64.1(6.4)^{c}	14.4(4.2)^{a}	15.3(2.9)^{a}	6.2 (2.1)^{a}	0.2(0.1)^{a}
Soil	48	41.8(3.0)^{b}	23.0(2.9)^{b}	25.9(3.9)^{c}	9.3 (1.5)^{b}	0.5(0.1)^{b}
Riverine	6	37.5(4.7)^{ab}	24.8(4.5)^{b}	28.5(1.7)^{c}	9.3 (1.9)^{b}	0.7(0.2)^{b}
IEMZ	5	37.4(5.8)^{ab}	24.0(2.0)^{b}	27.8(4.7)^{c}	10.8(1.2)^{b}	0.7(0.1)^{b}
OEMZ	4	33.6(3.0)^{a}	32.5(3.8)^{c}	19.6(4.7)^{ab}	14.3 (1.1)^{c}	1.0(0.1)^{c}
ITS	10	36.7(6.1)^{ab}	33.0(3.2)^{c}	21.4(6.8)^{ab}	8.9(1.9)^{b}	0.9(0.1)^{c}
NTS	32	34.6(4.1)^{a}	30.7(3.2)^{c}	24.2(4.8)^{bc}	10.7 (1.4)^{b}	0.9(0.1)^{c}

- Means (SD) within a column followed by the same letter are not significantly different at $p < 0.05$.
- ^{13}C NMR spectra chemical shift regions: alkyl C: 0-45 ppm; O-alkyl C: 45-110 ppm; aryl C: 110-160 ppm; carboxyl C: 160-180 ppm; A/O-A ratio: the ratio of alkyl C to O-alkyl C.
- Riverine: suspended particulate matter (SPM) collected from river; IEMZ: the SPM collected from inner estuarine mixing zone; OEMZ: the SPM collected from outer estuarine mixing zone; ITS: marine trap sediment influenced by flood plumes; NTS: marine trap sediment not influenced by flood plumes.

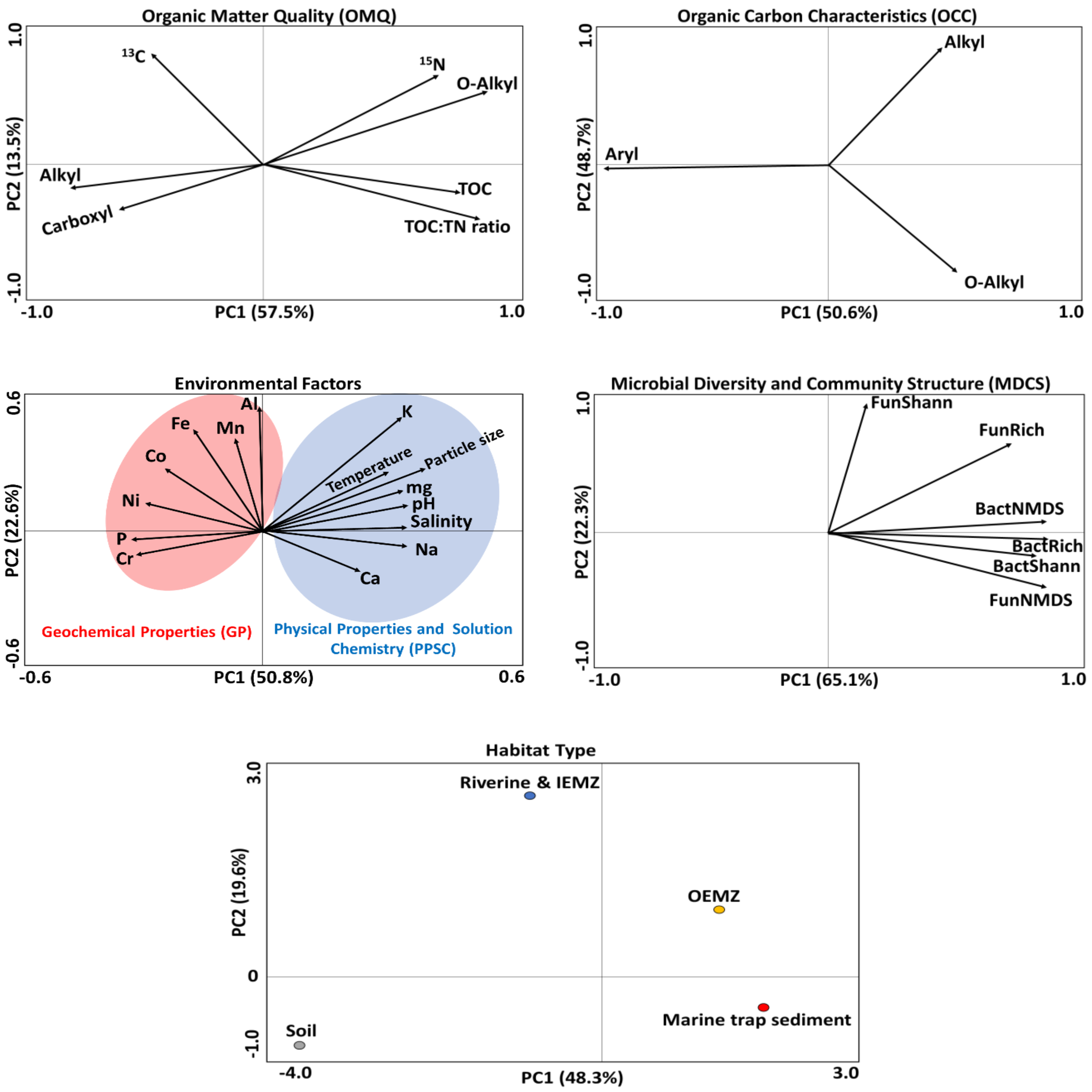

Fig. S4. The variation and correlation among biogeochemical variables across habitat types. These variables were used in the structural equation modelling. TOC: total organic carbon; TN: total nitrogen; BactRich and FunRich: bacterial and fungal richness, respectively; BactShann and FunShann: bacterial and fungal diversity Shannon Index, respectively; IEMZ: inner estuarine mixing zone; OEMZ: outer estuarine mixing zone.

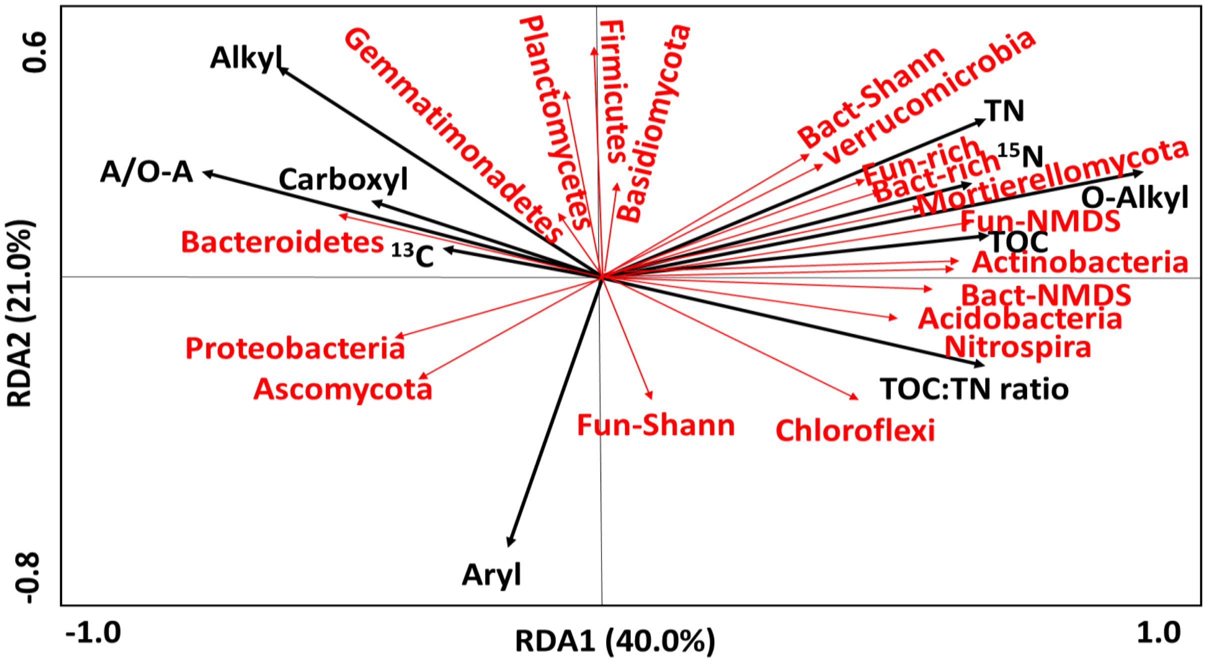

Fig. S5. Ordination biplot of Redundancy Analysis (RDA). This figure demonstrates the effects of microbial diversity and community structure (MDCS) on the characteristics of organic matter (OM) across soil, riverine, estuarine and marine habitats. The characteristics of OM included total organic carbon (TOC), total nitrogen (TN), TOC:TN ratio, $\delta^{13}C$, $\delta^{15}N$ and organic carbon functional groups (O-alkyl C, alkyl C, aryl C, carboxyl C) and alkyl C to O-alkyl C ratio (A/O-A ratio). Bacterial and fungal community composition, richness (BactRich and FunRich), diversity (Shannon Index - BactShann and FunShann) and community structure (BactNMDS and FunNMDS) of soil and suspended particulate matter were included in the RDA analysis to represent MDCS of soil, riverine, estuarine and marine habitats.

Table S2. Redundancy analysis (RDA) outputs showing the percentage of variation in organic matter (OM) characteristics across soil, riverine, estuarine and marine habitats explained by explanatory variables of microbial diversity and community structure (MDCS). The characteristics of OM included total organic carbon (TOC), total nitrogen (TN), TOC:TN ratio, $\delta^{13}C$, $\delta^{15}N$ and organic carbon functional groups (O-alkyl C, alkyl C, aryl C and carboxyl C) and alkyl C to O-alkyl C ratio (A/O-A ratio). Bacterial and fungal community composition, richness (BactRich and FunRich), diversity (Shannon Index - BactShann and FunShann) and community structure (BactNMDS and FunNMDS) of soil, riverine, estuarine and marine sediments were included in the RDA analysis to represent MDCS across habitats.

MDCS	Explains (%)	Pseudo-F	P-value
**BactNMDS**	**31.2**	**17.3**	**0.002***
Firmicutes	8.1	5	0.004*
Fun-Shann	5.0	4.1	0.004*
Mortierellomycota	5.8	4.2	0.006*
Verrucomicrobia	6.0	3.9	0.008*
Fun-rich	4.0	3.0	0.030*
Bact-Shann	2.4	2.2	0.070
Planctomycetes	2.4	2.1	0.086
Bacteroidetes	1.7	1.5	0.194
Basidiomycota	1.1	1.0	0.364
Gemmatimonadetes	1.1	1.0	0.406
Chloroflexi	1.1	0.9	0.438
Bact-rich	1.0	0.8	0.462
Proteocbactria	0.8	0.7	0.574
Actinobacteria	0.6	0.5	0.704
Nitrospira	0.5	0.4	0.790
Acidobacteria	0.5	0.4	0.792
Fun-NMDS	0.4	0.4	0.864
Ascomycota	0.2	0.1	0.988

*Statistically significant at $p < 0.05$.

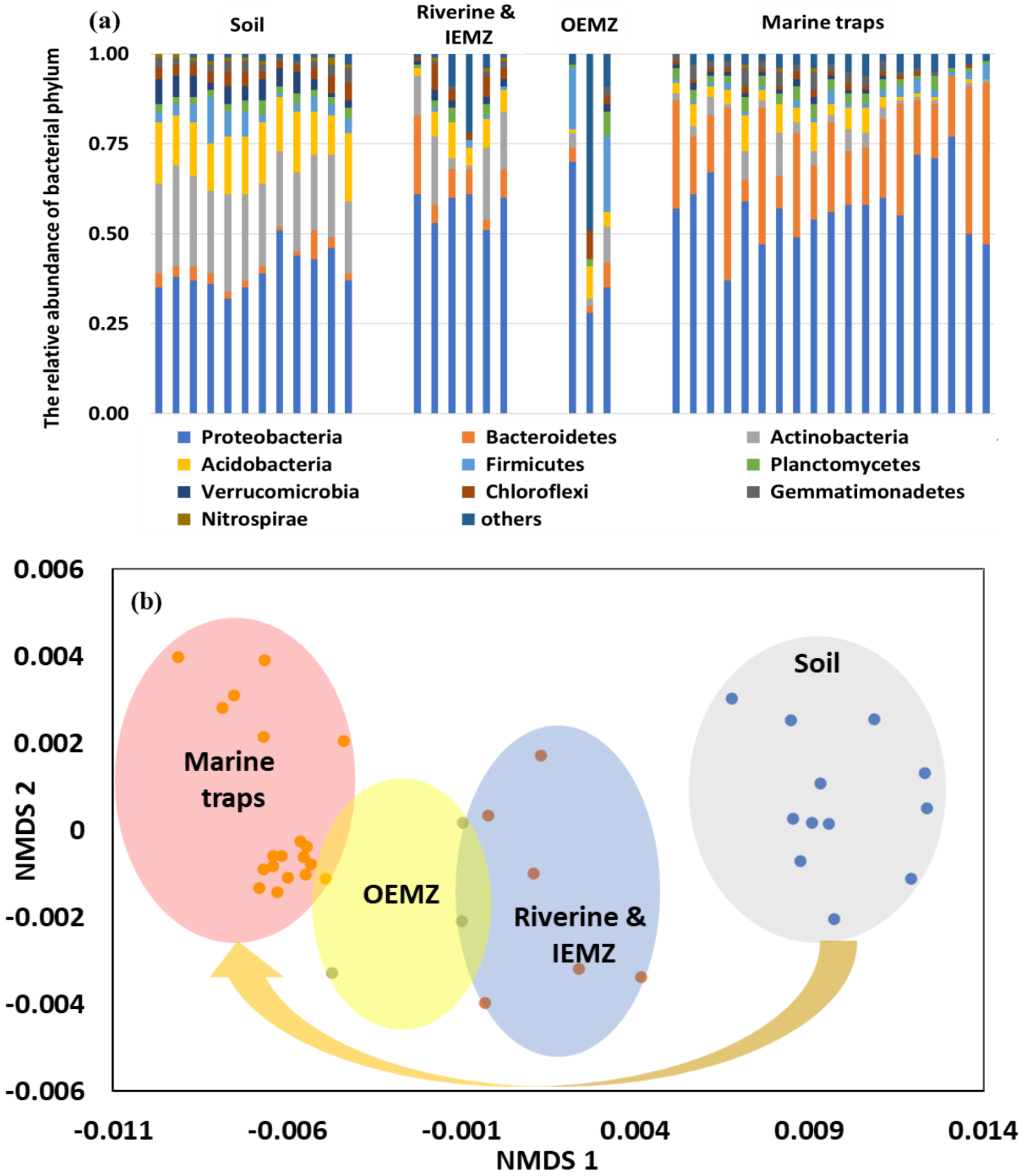

Fig. S6. Bacterial community composition and structure. This figure presents a) the relative abundance of bacterial community composition across habitats (each bar represents a sample); b) the overall patterns of bacterial community structure across habitats using non-metric multidimensional scaling (NMDS) analysis and Bray-Curtis dissimilarity distances. IEMZ: inner estuarine mixing zone; OEMZ: outer estuarine mixing zone.

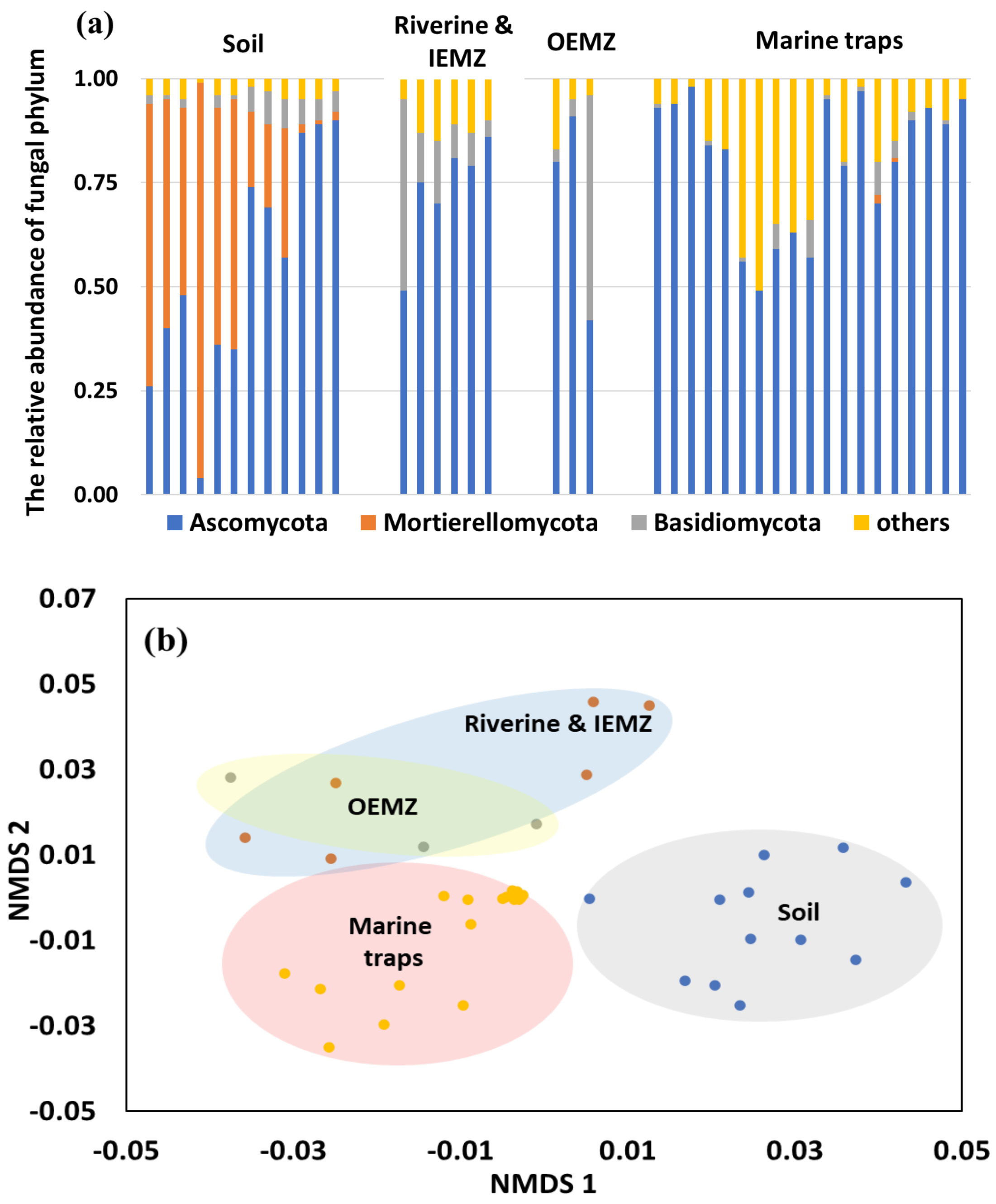

Fig. S7. Fungal community composition and structure. This figure presents a) the relative abundance of fungal community composition across habitats (each bar represents a sample); b) the overall patterns of fungal community structure across habitats using non-metric multidimensional scaling (NMDS) analysis and Bray-Curtis dissimilarity distances. IEMZ: inner estuarine mixing zone; OEMZ: outer estuarine mixing zone.

Origin of microbial communities in the marine environment

A Bayesian-based approach Sourcetracker2 was used to predict the contribution of different habitats (riverine, inner estuarine mixing zone (IEMZ) and outer estuarine mixing zone (OEMZ)) to bacterial and fungal communities associated with marine trap samples. The boxplots were generated in R, and Tukey Honest Significant Differences (TukeyHSD) was used to test the significance among different habitats.

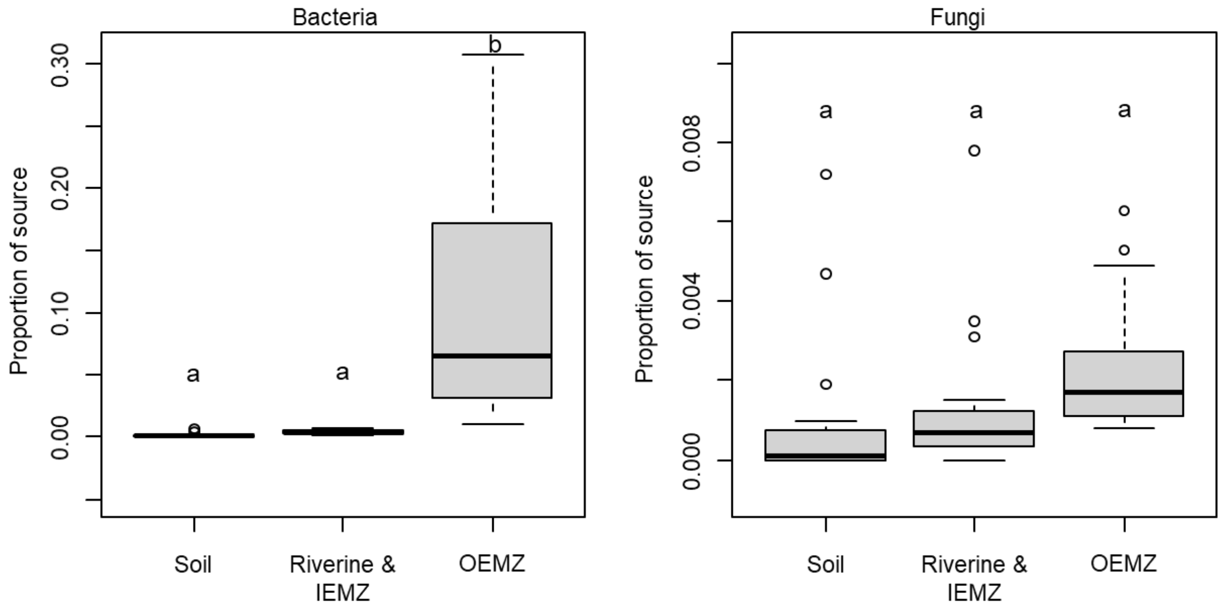

Fig. S8. The Origin of microbial communities in the marine environment. This figure shows the contribution of different habitats (riverine, inner estuarine mixing zone (IEMZ) and outer estuarine mixing zone (OEMZ)) to bacterial and fungal communities in marine trap samples. The average contributions of potential habitat sources are reported.

On average, 88.6% of the bacterial community associated with marine trap sediment was unique, while the remainder could be traced back to their potential habitat origin (Fig. S8). Of the three habitat sources, most bacteria associated with marine trap samples (10.8%) had OEMZ origins which was significantly ($p < 0.001$) higher than the contributions of soil, riverine and IEMZ combined (sum of which contributed only 0.6%). Results also showed that 99.54% of the fungi attached to marine trap sediments were unique, and only a small proportion could be traced back to the three habitat sources (0.09%-soil, 0.13%-riverine and IEMZ,

0.23%-OEMZ). These results show that microbial community composition and structure significantly change at the interface of freshwater and marine environments. Microbes in soil, riverine and IEMZ habitats comprise a very small proportion of microbial communities associated with marine trap sediment.

Table S3. Changes in the bacteria to fungi gene copy number ratio (bacteria:fungi ratio), bacterial and fungal richness (BactRich and FunRich), diversity (Shannon index - BactShann and FunShann) and community structure (BactNMDS and FunNMDS) across habitats.

Habitat	n	Bacteria:fungi ratio	BactShann	FunShann	BactRich	FunRich	Bacteria NMDS	Fungi NMDS
Soil	12	NA	8.6 (0.1)[c]	3.4 (0.8)[a]	8204 (812)[b]	1019 (212)[b]	0.0098 (0.0017)[d]	0.0258 (0.100)[b]
Riverine & IEMZ	6	2.4 (1.6)[a]	7.6 (0.7)[b]	5.1 (0.6)[b]	3126 (1835)[a]	960 (481)[b]	0.0014 (0.0017)[c]	-0.0104 (0.0205)[a]
OEMZ	3	5.5 (5.0)[ab]	6.9 (1.0)[a]	4.4 (1.0)[ab]	1613 (1119)[a]	656 (250)[ab]	-0.0022 (0.0021)[b]	-0.0176 (0.0185)[a]
Marine trap	19	8.6 (4.6)[b]	7.6 (0.2)[b]	3.0 (1.2)[a]	2904 (876)[a]	394 (253)[a]	-0.0063 (0.0072)[a]	-0.0102 (0.0093)[a]

- Means (SD) within a column followed by the same letter are not significantly different at $p < 0.05$.
- NA: not available
- IEMZ: inner estuarine mixing zone; OEMZ: outer estuarine mixing zone.
