# Supplementary material for: The origin of suspended particulate matter in the Great Barrier Reef
Source: Nat Commun. 2023 Sep 12;14:5629. doi: 10.1038/s41467-023-41183-z (PMC10497579; doi:10.1038/s41467-023-41183-z)
Supplement: Supplementary file 3 — Reporting Summary [file 41467_2023_41183_MOESM3_ESM.pdf]

nature portfolio

Corresponding author(s): Chengrong Chen

Last updated by author(s): Jul 29, 2023

Reporting Summary

Nature Portfolio wishes to improve the reproducibility of the work that we publish. This form provides structure for consistency and transparency in reporting. For further information on Nature Portfolio policies, see our Editorial Policies and the Editorial Policy Checklist.

Statistics

For all statistical analyses, confirm that the following items are present in the figure legend, table legend, main text, or Methods section.

n/a	Confirmed	
☐	☒	The exact sample size (*n*) for each experimental group/condition, given as a discrete number and unit of measurement
☐	☒	A statement on whether measurements were taken from distinct samples or whether the same sample was measured repeatedly
☐	☒	The statistical test(s) used AND whether they are one- or two-sided<br>*Only common tests should be described solely by name; describe more complex techniques in the Methods section.*
☐	☒	A description of all covariates tested
☐	☒	A description of any assumptions or corrections, such as tests of normality and adjustment for multiple comparisons
☐	☒	A full description of the statistical parameters including central tendency (e.g. means) or other basic estimates (e.g. regression coefficient) AND variation (e.g. standard deviation) or associated estimates of uncertainty (e.g. confidence intervals)
☒	☐	For null hypothesis testing, the test statistic (e.g. *F*, *t*, *r*) with confidence intervals, effect sizes, degrees of freedom and *P* value noted<br>*Give P values as exact values whenever suitable.*
☒	☐	For Bayesian analysis, information on the choice of priors and Markov chain Monte Carlo settings
☒	☐	For hierarchical and complex designs, identification of the appropriate level for tests and full reporting of outcomes
☒	☐	Estimates of effect sizes (e.g. Cohen's *d*, Pearson's *r*), indicating how they were calculated

Our web collection on statistics for biologists contains articles on many of the points above.

Software and code

Policy information about availability of computer code

Data collection	No commercial, open source and custom data was used.
Data analysis	No commercial, open source and custom code was used.

For manuscripts utilizing custom algorithms or software that are central to the research but not yet described in published literature, software must be made available to editors and reviewers. We strongly encourage code deposition in a community repository (e.g. GitHub). See the Nature Portfolio guidelines for submitting code & software for further information.

Data

Policy information about availability of data

All manuscripts must include a data availability statement. This statement should provide the following information, where applicable:

- Accession codes, unique identifiers, or web links for publicly available datasets
- A description of any restrictions on data availability
- For clinical datasets or third party data, please ensure that the statement adheres to our policy

All 16S rRNA gene and ITS fragment sequences were deposited in the Sequence Read Archive (SRA) with the accession number PRJNA690602. The data for bioavailability and chemical composition of the organic component of soils and SPM from the catchment to reef project (NESP TWQ 5.8) has been submitted to e-Atlas data catalogue (Australian Institute of Marine Science) and can be accessed at https://eatlas.org.au/data/uuid/3dadf670-f9b1-44d8-bce0-a5aeddf2c20a.

Research involving human participants, their data, or biological material

Policy information about studies with human participants or human data. See also policy information about sex, gender (identity/presentation), and sexual orientation and race, ethnicity and racism.

Reporting on sex and gender	NA
Reporting on race, ethnicity, or other socially relevant groupings	NA
Population characteristics	NA
Recruitment	NA
Ethics oversight	NA

Note that full information on the approval of the study protocol must also be provided in the manuscript.

Field-specific reporting

Please select the one below that is the best fit for your research. If you are not sure, read the appropriate sections before making your selection.

☐ Life sciences ☐ Behavioural & social sciences ☒ Ecological, evolutionary & environmental sciences

For a reference copy of the document with all sections, see nature.com/documents/nr-reporting-summary-flat.pdf

Ecological, evolutionary & environmental sciences study design

All studies must disclose on these points even when the disclosure is negative.

Study description	In this study we provide multiple lines of evidence to unravel that a considerable proportion of the terrestrially-derived suspended particulate matter (SPM) is degraded in the riverine and estuarine mixing zones before it is transported further offshore into the marine environment and the Great Barrier Reef.
Research sample	A total of 35 soil samples were collected to characterise the four key land uses. The SPM were collected from freshwater sections (riverine), inner estuarine mixing zone (IEMZ) and outer estuarine mixing zone (OEMZ) of the Johnstone, Tully and Burdekin Rivers. In general, the separation of these estuarine mixing zones are related to changes in SPM concentration and salinity and are broadly related to primary and secondary colour class water types established in remote sensing studies.
Sampling strategy	The selected catchments for sampling varied in their land use, catchment area, geographical location, vegetation type, geological structure, and climate, thereby capturing a wide range of terrestrially-derived SPM with various chemical and isotopic signatures/ endmembers and microbial diversity and community structure.
Data collection	For data collection, all collected samples were misanalysed for bio geochemical structures in laboratory.
Timing and spatial scale	The GBR stretches over a significant area including inshore, mid-shelf and offshore regions, however, in this study the marine traps were installed within the inner GBR shelf to capture sediment resuspension events (dry season deployment) as well as the influence of SPM delivered through the riverine flood plumes (during the wet season deployments). Soil samples were collected across Wet and Dry Tropics regions of the GBR.
Data exclusions	No data was excluded.
Reproducibility	All data collections and laboratory analyses have been conducted with replications to confirm the reproducibility.
Randomization	Samples were randomly selected across the catchments of the GBr including both Wet Tropics and Dry Tropic regions
Blinding	Blinding was not relevant to this study.

Did the study involve field work? ☒ Yes ☐ No

Field work, collection and transport

Field conditions	All the SPM samples (riverine, IEMZ and OEMZ) were collected during the flood plume events during the wet seasons (January to March) of 2017, 2018 and 2019 to capture pulsed inputs of SPM during flood events with the potential to reach out well beyond the inner GBR and into the mid-shelf area.
Location	An array of three to four marine traps was established at seven sites off the coast from the Burdekin catchment in Cleveland Bay

	(e.g., Middle Reef, Cleveland Bay, Orchard Rocks, Geoffrey Bay) and Halifax Bay (e.g., Orpheus, Pelorus and Havannah Islands) to capture the SPM exposure in the Dry Tropics region including the influence of the Burdekin River flood plume (Fig. 1). Similarly, the Dunk Island marine trap site captured the SPM originating from the Wet Tropics region including the flood plume from the Tully River.
Access & import/export	The field sediment traps, and instrument loggers were deployed under permits G17/38148.1 and G16/38774.1 issued by the Great Barrier Reef Marine Park Authorities.
Disturbance	Now disturbance was made during sampling.

Reporting for specific materials, systems and methods

We require information from authors about some types of materials, experimental systems and methods used in many studies. Here, indicate whether each material, system or method listed is relevant to your study. If you are not sure if a list item applies to your research, read the appropriate section before selecting a response.

Materials & experimental systems

n/a	Involved in the study
☒	☐ Antibodies
☒	☐ Eukaryotic cell lines
☒	☐ Palaeontology and archaeology
☒	☐ Animals and other organisms
☒	☐ Clinical data
☒	☐ Dual use research of concern
☒	☐ Plants

Methods

n/a	Involved in the study
☒	☐ ChIP-seq
☒	☐ Flow cytometry
☒	☐ MRI-based neuroimaging
